# Results of Nucleos(t)ide Analog Treatment Discontinuation in Hepatitis B e-Antigen-Negative Chronic Hepatitis B: NUCSTOP Study

**DOI:** 10.5152/tjg.2024.23463

**Published:** 2024-01-01

**Authors:** Sercan Kiremitçi, Koray Koçhan, Gülseren Seven, Elmas Biberci Keskin, Gülay Okay, Yasemin Akkoyunlu, Meliha Meriç Koç, Bilge Sümbül, Hakan Şentürk

**Affiliations:** 1 Department of Gastroenterology, Bezmialem University School of Medicine, İstanbul, Turkey; 2 Department of Infectious Diseases and Clinical Microbiology, Bezmialem University School of Medicine, İstanbul, Turkey; 3 Department of Medical Microbiology, Bezmialem University School of Medicine, İstanbul, Turkey

**Keywords:** Chronic hepatitis B, HBeAg-negative, HBsAg loss, lamivudine resistance, nucleos(t)ide analog discontinuation

## Abstract

**Background/Aims:**

This study aims to investigate the effects of nucleos(t)ide analogs (NAs) discontinuation in eligible patients in accordance with the Asian Pacific Association for the Study of the Liver hepatitis B guideline and the factors affecting clinical and virological relapses.

**Materials and Methods:**

In this prospectively designed study, hepatitis B e antigen (HBeAg)-negative chronic hepatitis B patients who were followed up between 2012 and 2019 were evaluated and 57 patients were included. All participants enrolled the study were HBeAg-negative status at NA initiation.

**Results:**

The median age of the patients was 49 (29-72) years and 24 (42%) were females. The median treatment duration was 96 (36-276) months and patients were followed for a median duration of 27 months. Sixteen patients had a previous history of NA switch, and thirteen of these patients had a history of lamivudine resistance. Thirty-eight of 57 patients (66%) developed an elevated hepatitis B virus deoxyribonucleic acid level of >2000 IU/mL at least once, defined as virological relapse and 23 (60%) of them, experienced clinical relapse. Thirty-one of 57 patients were re-treated during the follow-up, and hepatitis B surface antigen (HBsAg) loss occurred among 4 (7%) patients. All patients who experienced HBsAg loss had a history of lamivudine resistance (*P* = .002).

**Conclusion:**

Despite receiving NAs suppression therapy for a long time, HBsAg loss occurs rarely. Although it was not life-threatening, most patients experienced relapses and treatment should be restarted. In our study, whether it is a coincidence that all patients with HBsAg loss are patients in whom NAs are used sequentially due to lamivudine resistance is an issue that needs to be further investigated.

**Main Points**

- Discontinuation of nucleos(t)ide analogs, before HBsAg loss is investigational. Because, despite many studies, comprehensive and specified criteria for treatment discontinuation have not been defined yet.
- The follow-up of relapses and determining the factors affect relapses after nucleos(t)ide analogs discontinuation are important for safe treatment discontinuation.
- Older age, female gender, higher pretreatment HBV-DNA, ALT, and HAI score predict the relapses in our study.
- The main outcome of the study is that all patients with HBsAg loss were the ones with previous lamivudine resistance that needs to be further investigated.

## Introduction

Chronic hepatitis B (CHB) still maintains its importance all over the world despite effective vaccination programs. The Advisory Committee on Immunization Practices (ACIP) and the World Health Organization (WHO) strongly recommend vaccination as the most efficient tool for prevention of hepatitis B virus (HBV) infection;^1^ however, hepatitis B is one of the most important reasons of end-stage liver diseases and affects approximately 400 million patients worldwide.^2^ It is, indeed, the leading cause in underdeveloped countries and annually over the 750 000 patients die from HBV-related chronic liver diseases.^3^ The implementation of safe and potent drugs has affected positively the outcome of patients with CHB over the last 15 years.^4^ Especially, widespread use of potent, first line nucleos(t)ide analogs (NAs), such as entecavir (ETV) and tenofovir, has provided extended inhibition of HBV replication in almost all compatible patients with CHB, improvement of necroinflammation and fibrosis, sometimes reversion of histological cirrhosis and protection from advanced liver disease.^5,6^ Since NAs act on the late phase of the viral cycle and do not directly effect on the covalently closed circular DNA (cccDNA), patients rarely experience hepatitis B surface antigen (HBsAg) loss.^7^ Previous studies have demonstrated that the annual rate of functional cure (HBsAg loss) is almost 0.5%-2.3%.^8^ Therefore, in current clinical practice, most patients with CHB needs to continue NA treatment throughout their lives once the treatment has started. As a result, an indefinite duration of NA treatment raises concerns about the economic burden, the risk of developing resistance, adherence, and adverse events. The recently updated CHB guidelines are increasingly recommending the possibility of NA discontinuation in selected patients.

We designed a prospective, single-center and observational study to reveal the effects of NA discontinuation in HBeAg-negative CHB patients in an exclusively genotype D area.

## Materials and Methods

### Patients and Study Design

This is a prospective, single-center, and observational study. The files of patients with CHB followed up in the Gastroenterology and Infectious Diseases departments in Bezmialem Foundation University Hospital between 2012 and 2019 were examined and 57 eligible and agreed patients were included. After eligible patients were identified, the treatment was discontinued and the patients have been started to follow-up prospectively. NA treatment was discontinued in accordance with the treatment discontinuation recommendations in the Asian Pacific Association for the Study of the Liver (APASL) hepatitis B guideline.^2^ According to APASL guideline, NA treatment can be withdrawn in patients with HBeAg-negative CHB after treatment for at least 2 years with undetectable HBV-DNA documented on 3 separate occasions, 6 months apart.^2^ The reason why we preferred the APASL hepatitis B guideline in this study is that it provides clearer criteria for treatment discontinuation. The EASL hepatitis B guideline specifically mentions about the accumulating data from Asian studies, regarding the discontinuation of NA treatment in HBeAg-negative patients. We thought that having more results related to Asian studies would be beneficial in terms of study safety. Liver biopsy had been performed to all patients before NA treatment was started and and biopsy results were obtained from the patient record system with permission.

The patients were followed up with predetermined visits. At each visit with biochemical tests [aspartate transaminase (AST), alanine transaminase (ALT), total and direct bilirubin, albumin, urea, creatinine, and electrolytes], virological panel (HBsAg, HBV-DNA, HBeAg, anti-HBe), INR, alpha-fetoprotein (AFP) measurement, and complete blood count were done. Besides, screening for hepatocellular carcinoma (HCC) using transabdominal ultrasonography (TAUS) was performed every 3-6 months. Written informed consent form was obtained from all participants before participating in the study.

This study was approved by the decision of Bezmialem Foundation University Ethics Committee, which functions in accordance with the Declaration of Helsinki, dated February 6, 2019. (Reference number: 71306642-050.01.04). Written informed consent was obtained from the patients who participated in this study.

### Inclusion and Exclusion Criteria

The patients at or over 18 years old, receiving NAs therapy for HBeAg-negative hepatitis B, and undetectable or negative HBV-DNA for at least 2 years with normal ALT were included. The exclusion criteria were presence of cirrhosis at the time of NA discontinuation; coinfected with human immunodeficiency virus (HIV), hepatitis C, hepatitis D, immunocompromised status; with malignancy, and liver transplanted patients.

### Definitions and Retreatment Decision

Relapses were categorized as virological, biochemical, or clinical. HBV-DNA results were calculated as log_10_ IU/mL, except this only the quantitative value was taken as reference for the determination of relapse. Virological relapse was defined as HBV-DNA is above 2000 IU/mL at one time of off-treatment follow-up. Biochemical relapse was defined as ALT >2× upper limit of normal (ULN) at one time of off-treatment follow-up, and clinical relapse was defined as HBV-DNA >2000 IU/mL and ALT >2× ULN combined.^9^ Sustained response was defined as HBV-DNA < 2000 IU/mL and persistently normal level of ALT at off-treatment follow-up.^10^ Findings considered indicating cirrhosis on imaging include nodular liver parenchyma, splenomegaly, or portal vein diameter of >16 mm.^11^ Consolidation treatment duration was defined as the treatment time after undetectable HBV-DNA and ALT normalization was achieved for HBeAg-negative CHB patients.^2^ LAM resistance was defined as at least 0.17 log_10_ IU/mL raise or virologic breakthrough of HBV-DNA in at least 2 visits under LAM treatment.^12^ ALT > 10× ULN or ALT > 5× ULN + total bilirubin >2 mg/dL and/or prothrombin time prolongation after discontinuation of treatment have been considered as a severe flare in previous studies.^13^ We used the same criteria for defining severe flare. During the follow-up period after treatment discontinuation, we used the same re-treatment criteria and started treatment again with the consensus of the following physician and patient. In some patients, treatment has been restarted without our initiative.

### Laboratory Assays

Biochemical tests (ALT, AST, bilirubin, etc.) were determined using the photometric method (Abbott Park, Chicago, Illinois, USA), and hormone analysis (AFP) was done using the electrochemiluminescence method (Abbott Architect I 2000 SR). Prothrombin time was measured by Mechanical Clot Detection using a coagulation autoanalyzer; AMAX 200 Amelung, Trinity Biotech, Ireland. HBV-DNA quantitation was done by in vitro polymerase chain reaction (PCR) assay in patients’ serum by m2000sp device E-Series (Abbott) with Abbott Real Time HBV kit. HBsAg Qualitative II Reagent kit, Alinity I HBeAg and Anti-HBe Reagent kit were used for the assessment of HBsAg, HBeAg, and anti-HBe. Alinity (Abbott) device was used to determine serum levels of antibodies.

### Statistical Analysis

In this study, statistical analysis was performed using the software of NCSS (Number Cruncher Statistical System) 2007 Statistical Software (Kaysville, Utah, United States) and the Statistical Package for Social Sciences (SPSS) version 25.0 (IBM Corp., Armonk, NY, USA). In addition to the descriptive statistical analysis (mean, SD, median, range); we used the independent t-test for the comparison of the normally distributed variables between paired groups, and the Mann–Whitney *U*-test for the comparison of the non-normally distributed variables between the paired groups, while the chi-square test (Yates correction) and Fisher exact test for the comparison of qualitative data. Box plot was utilized to visualize the distribution of HBV-DNA (log_10_) data by week. Logistic regression analysis was conducted to investigate the factors affecting the variables of virological and clinical relapses. Receiver operating characteristic (ROC) curve analysis was carried out to predict HBV-DNA level concerning virological relapse and liver biopsy Knodell’s histological activity index (HAI) score in patients with CHB. As a result of ROC curve analysis, HBV-DNA level, and HAI score cutoff values in patients with virological relapse were determined using corresponding sensitivity and specificity. Area under receiver operating characteristic curve (AUROC) value was computed with a confidence interval (CI) of 95%. The results were considered significant at *P* < 0.05.

## Results

In total, 57 patients were enrolled to the study. All patients were HBeAg-negative at the start of NA treatment. Liver biopsy had been performed to all patients before the initiation of NA treatment. The median liver biopsy Ishak fibrosis scores of the patients before the start of NA treatment was 2 (range 0-3) and the median Knodell HAI score was 6 (range 2-13). At the treatment discontinuation, HBV-DNA levels of all patients were below the lower limit of quantification. The median age was 49 years (range 29-72) and 24 (42%) were females. Patients were followed up for a median duration of 27 months (range 24-30 months). The median treatment duration was 96 months (range 36-276 months), while the median duration of consolidation was 88 months (range 31-273 months). At the discontinuation, 32 patients were receiving tenofovir and 17 entecavir, while 8 were receiving LAM, adefovir, or telbivudine. Sixteen (28%) patients NA therapy was switched in the period before discontinuation of treatment. Thirteen of these 16 patients had a history of treatment switch due to LAM resistance. Of the 16 patients whose treatment were switched; thirteen experienced a virological breakthrough or partial virological response during LAM treatment. One had high persistence of liver enzymes over 6 months despite treatment, 1 developed impotence and 1 patient developed acute coronary syndrome. These findings were not considered as a direct drug side effect. Coincidentally, they were determined when the NA treatment had been changed by physician.

Pretreatment median-range HBV-DNA (log_10_) level was 5.31 (3.47-8.95) log_10_ IU/mL and pretreatment median-range ALT level was 43 U/L (12-154 U/L). The clinical characteristics of the patients at NA cessation (n = 57) are depicted in Table 1.

In 2 patients, ALT values were within the normal range at the time of NA initiation. In the pretreatment follow-up, these patients ALT values were intermittently high and within the normal range. In addition, one of these patients was over 40 years old and had a family history of hepatocellular cancer. For this reason, NA initiation values were taken as reference.

**Figure 1.**
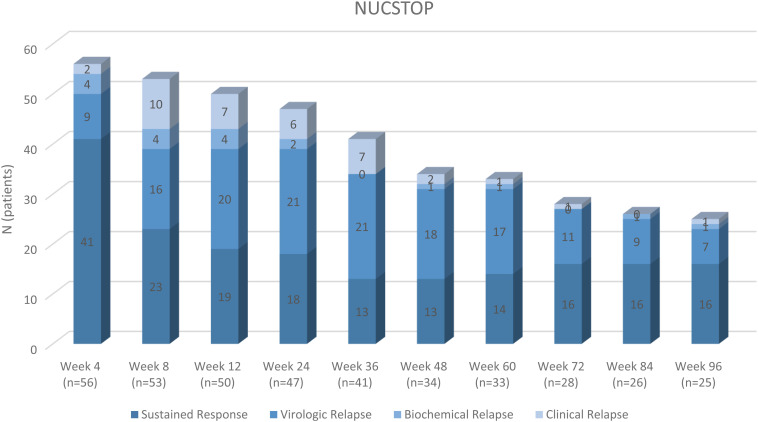
The status of the patients whose treatment discontinued and followed up untreated according to weeks.

**Figure 2.**
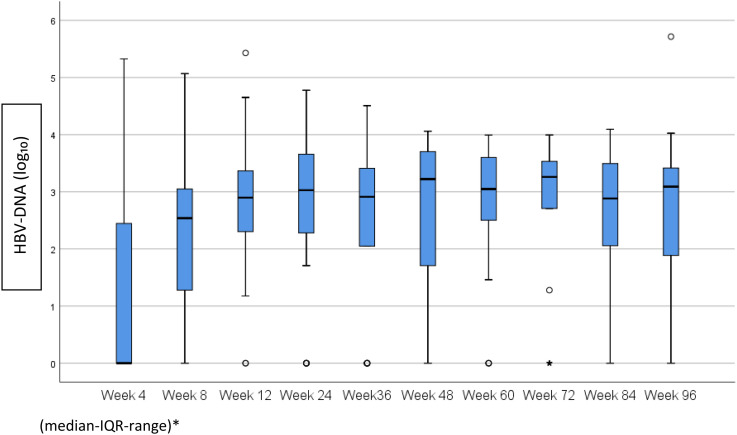
HBV-DNA (log_10_) levels of the patients followed up untreated according to weeks. HBV-DNA, hepatitis B virus deoxyribonucleic acid.

The status of the patients whose treatment discontinued and followed up untreated is shown by weeks with a column chart in Figure 1. In the follow-up process, 31/57 (54%) patients were re-treated, and 1 patient gave up the follow-up. HBV-DNA (log_10_) levels (median IQR range) of the untreated patients at the follow-up are shown with a box plot graphic (Figure 2).

During the follow-up, 38 patients (66%) developed a virologic relapse, of whom 23 experienced clinical relapse. In univariate and subsequent multivariate logistic regression analysis, biochemical relapse [odds ratio (OR) 0.092; 95% CI 0.015-0.571; *P* = .01], female gender (OR 16.126; 95% CI 1.213-214.321; *P* = .035), and pretreatment HBV-DNA (log_10_) level (OR 0.375; 95% CI 0.170-0.828; *P* = .015) were revealed as independent risk factors for virologic relapse. Pretreatment liver biopsy HAI score was not an independent predictor for virologic relapse in logistic regression analysis, although it was different between the patients groups who did not relapse and the ones who did using the *t*-test (5.42 ± 1.83 vs.7.47 ± 2.42, *P* = .002)**. **Only,in the univariate logistic regression analysis, older age (OR 1.096; 95% CI 1.025-1.172; *P* = .008), higher pretreatment HAI score (OR 1.344; 95% CI 1.044-1.731; *P* = .022), higher pretreatment HBV-DNA (log_10_) (OR 1.738; 95% CI 1.075-2.811; *P* = .024) and higher pretreatment ALT (OR 1.028; 95% CI 1.004-1.053; *P* = .022) levels were significantly associated with clinical relapse. No significant result was found in the multivariate logistic regression analysis.

In our study, we tried to determine discriminating values that could predict virological relapse and constructed ROC curve analysis. The ROC curve analysis of the pretreatment HBV-DNA levels of patients with virological relapse demonstrated, the ideal cutoff value for the pretreatment HBV-DNA (log_10_) level predicting virological relapse was 4.72 (log_10_) IU/mL (AUROC 0.735, *P* = .004). Sensitivity of this score was 81.6% and specificity 68.4%. Besides, the ROC curve analysis of HAI score of pretreatment liver biopsy of patients with virological relapse demonstrated, the ideal cutoff value score that predicts virological relapse, was 6.50. Sensitivity of this score was 57.9% and specificity, 84.2% (AUROC 0.751, *P* = .002).

At the end of the follow-up period, there were 25 patients’ treatment has not been restarted, while in 17 patients, serum HBV-DNA was below 2000 IU/mL and it was over this value in 8 patients. Re-treatment has been started in 31 patients. The persistence of virological relapse (HBV-DNA >2000 IU/mL) at the end of the follow-up period was correlated with high body mass index (*P* = .041) and liver biopsy HAI score (*P* = .022) in univariate logistic regression analyses, as shown in Table 2.

**Supplementary Figure 1.**
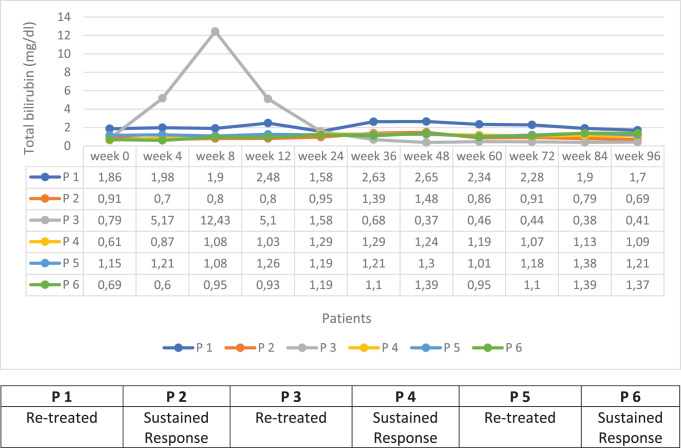
The total bilirubin course of the patients with elevated bilirubin level in the follow-up.

Throughout the follow-up period, elevation in serum bilirubin was detected in 6 patients, and the highest total bilirubin level detected was 12.4 mg/dL. Three of these patients have still been followed without treatment, and the other 3 have been re-treated. The HBV-DNA levels of the patients who did not re-treated were 1466 IU/mL, 262 IU/mL, and 371 IU/mL, respectively, and all of them were in the sustained response status. Hepatic decompensation has not been experienced in any of the patients, and no prolongation of prothrombin time was detected. The total bilirubin courses of the patients up to 96-weeks follow-up are presented in Supplementary Figure 1.

In order to investigate the factors affecting LAM resistance, statistical analysis was performed on patient groups with and without LAM resistance, and no significant difference was found between these 2 groups, except HBsAg clearance rate and FIB-4 score. There was no significant difference between the parameters such as duration of NAs exposure, duration of consolidation, age, gender, degree of fibrosis, and pretreatment HBV-DNA level in patient groups with and without LAM resistance. The FIB-4 score was found to be significantly higher in the group with LAM resistance (*P* = .042). The results are shown in Table 3.

Four patients lost HBsAg after NA therapy discontinuation, and the cumulative functional cure (HBsAg loss) rate was 7%. Significant titer of anti-HB positivity was detected in 2 of the 4 patients. There was no difference between treatment agents regarding HBsAg loss. Among the patients who achieved HBsAg loss, 2 experienced transient virological relapse (HBV-DNA >2000 IU/mL) and 1 experienced clinical relapse before HBsAg clearance. Participants who achieved HBsAg clearance, 3 of them were male and 1 female, with a median age of 53 years (range 40-70 years). All patients who achieved HBsAg clearance had a history of previous LAM resistance (*P* = .002) (Fisher’s exact test). Total treatment durations were 144, 84, 144, and 96 months for each patient, respectively. After the emergence of resistance, 3 had been treated with tenofovir and 1 with ETV. Also, 1 patient had a history of PEG-IFN treatment. Pretreatment median HBV-DNA (log_10_) level was 4.99 (range 3.53-7.17 log_10_ IU/mL) and median ALT level was 43 U/L (range 32-52 U/L). Their pretreatment liver biopsy Ishak fibrosis scores were 0, 3, 2, and 2.

Thirty-one patients have been re-treated with the previously used antiviral agents. The maximal ALT flare recorded was 1313 U/L. In all patients, normal ALT and undetectable HBV-DNA levels were achieved after re-treatment. HBsAg clearance did not happen in any patient during re-treatment. During the study and follow-up period, liver decompensation or hepatocellular cancer was not detected in any patient.

## Discussion

Discontinuation of NAs before HBsAg loss is investigational because, despite many studies, comprehensive and specified criteria for treatment discontinuation and relapses have not been defined yet. Therefore, studies on this topic still maintain their investigational status. In our study, 57 HBeAg-negative CHB patient’s treatment was discontinued and enrolled in accordance with the treatment discontinuation criteria stated in the APASL hepatitis B guideline. Our study group was HBeAg-negative, and guidelines set various treatment discontinuation criteria for HBeAg-negative patients. The European Association of the Study of Liver Disease (EASL) stated that this would be possible after at least 3 years of virological suppression, while APASL stated that the treatment could be discontinued in patients who received NAs therapy for at least 2 years and had undetectable HBV-DNA levels in the serum 3 times with an interval of 6 months. On the other hand, The American Association for the Study of Liver Diseases (AASLD) recommends that antiviral therapy can be discontinued only in patients with HBsAg clearance.^2,6,14^

In a median 27 months follow-up of our 57 HBeAg-negative participants, HBsAg clearance was achieved in 4 (7%). This rate and patient characteristics are similar to the study group of Buti et al.^15^ It is well documented that after HBsAg loss, hepatitis B surface antibody (anti-HBs) positivity provides a more effective immune control,^16^ and in our study, anti-HBs was positive at a significant titer in 2 of the 4 patients with HBsAg clearance. Likewise, in another study investigating the impact of discontinuation of treatment on HBsAg clearance, the rate of functional cure was reported as 19% in group who discontinued treatment, while none lost who did not for 144 weeks.^17^

Throughout the follow-up period, virological relapse was detected in 66% of our patients. Our virological relapse rates are like those in previous studies.^18^ When we examined the factors affecting virological relapse, female gender and higher pretreatment HBV-DNA levels appeared significant in multivariate logistic regression analysis. Hitherto studies did not report such a correlation.

Transient virological relapse occurred in 2 of the 4 patients with HBsAg clearance in our group. Previous studies have revealed that HBV-specific T cells are dormant by long-term NA treatment, but after discontinuation of therapy, the host immune system is re-exposed to replicating viral antigens and respond appropriately, as it is in acute hepatitis B.^19,20^ In another study supporting this hypothesis, NAs were discontinued in 1 of the 2 groups and continued in the other; albeit a high rate of virological relapse occurred in the discontinued group, HBsAg loss occurred at a higher rate.^21^ Höner Zu Siederdissen et al^20^ support the hypothesis that immune system reactivation after NA discontinuation also allows better viral control, suggesting that it may predict HBsAg loss after HBV-DNA reactivation. These mechanisms may explain the contribution of virologic relapse to a possible functional cure.

Chi et al stated that consolidation therapy over 3 years before NA discontinuation reduced the risk of virologic relapse in both HBeAg-positive and HBeAg-negative groups.^22^ However, we did not find a statistically significant correlation between the duration of consolidation treatment and virologic relapse (*P* = .072). The mean consolidation treatment duration of our patients was above the recommended durations in the guidelines; however, our virological relapse rates were still high after discontinuation of treatment. There are other studies suggesting that the consolidation therapy duration is not predictive for virologic relapse.^23^ In this regard, the contribution of longer consolidation therapy to effective virological suppression is controversial.

In previous studies, many quantitative parameters that could predict sustained virological response or virological relapse during follow-up without treatment were studied. Seto W-K et al reported that the risk of off-treatment HBV reactivation is lower in patients with low HBV-RNA and quantitative HBsAg (qHBsAg) levels. Cutoff values were calculated as 44.6 U/mL for HBV-RNA and 10 IU/mL for qHBsAg level.^24^ We did not measure these. However, we determined cutoff values for pretreatment HBV-DNA and HAI score predicting virological relapse in ROC curve analysis. We believe that the cutoff values determined by the studies will be effective in the decision to NA discontinuation.

Another important point to be clarified is the optimal timing of re-treatment in case it is supposed to be necessary. Currently, the EASL guideline has determined the re-treatment strategy in treatment-discontinued patients in parallel with HBV-DNA >2000 IU/mL and ALT > ULN.^6^ Various criteria and approaches have been underscored in studies on this issue. In some studies, re-treatment was started when HBV-DNA rise above 2000 IU/mL, regardless of ALT elevation.^24,25^ On the other hand, in Papatheodoridis et al’s^26^ study, a clear pattern was determined for re-treatment. They recommended that severe flares such as ALT >10× ULN or ALT >5× ULN plus bilirubin >2 mg/dL (predominantly direct) and/or prolongation of prothrombin time should be treated again. Also, ALT > ULN and HBV DNA >2000 IU/mL persistently for at least 3-6 months which described as persistent mild to moderate liver disease activity should be treated.^13^ In an Asian study, merely 11.7% of the patients had ALT, more than doubled, although 91.4% of the patients developed virological relapse. Since most of the patients in this study group were re-treated, the rate of patients with the functional cure was remarkably low.^27^ As Hadziyannis et al^10^ stated in their pivotal study, when adefovir treatment of 33 HBeAg-negative patients was discontinued, all patients experienced virological relapse and 76% experienced biochemical relapse from the first month onward. However, in this study, the patients did not restart the treatment as soon as the relapse occurred and continued to be followed up, resulting in HBsAg loss in 39% of the patients. In this regard, it makes sense to assume that isolated virologic relapse without the risk of decompensation is not an appropriate indication for restarting therapy.

Currently, close follow-up after treatment discontinuation seems to be the most crucial point to detect potential adverse events and take immediate measures. Given the studies in which virological and biochemical relapses were observed more frequently in HBeAg-negative patients after discontinuation of treatment,^26^ our HBeAg-negative patient group was followed up very closely, especially in the first 3-6 months, and additional follow-ups were scheduled when necessary.

Whether the cost of strict follow-up or the cost of NAs medications outweighs is a matter of debate. Other issues that need to be addressed in further studies are when to initiate retreatment, and to determine which relapse is beneficial, and which poses the risk of decompensation. In a published systematic review, incidence of hepatic decompensation after NA discontinuation was calculated between 0.8 and 3%,^26^ which is acceptable.

Like many other studies, we did not set our re-treatment criteria very strictly; we followed intended not to re-treat except for flares (ALT >10× ULN, total bilirubin >3 mg/dL and/or prothrombin time prolongation) that carry the risk of decompensation. In the systemic review published by Papatheodoridis et al,^26^ it was reported that rapid suppression was achieved with retreatment in patients who developed flare after treatment discontinuation. This was also the case in our patient group; no adverse events occurred.

Pivotal result of our study was that HBsAg loss was significantly higher in the group with a history of LAM resistance (*P* = .002) (Fisher exact test). When we compared the characteristics of the patients who had developed LAM resistance against the ones who did not, the only significant difference was in FIB-4 score that the score of the former group was higher. The significant correlation between a history of LAM resistance, and loss of HBsAg, in the HBeAg-negative patient group, is a noteworthy issue that needs to be further investigated.

The first main limitation of our study was that HBV genotype determination was not performed, as most previous studies did not reveal a significant association between genotype determination and relapses in the treatment-free follow-up period.^18,28^ The lack of quantitation of HBsAg is the second main limitation. When evaluated in terms of reactivation, Nirmala et al reported that there was no significant correlation was observed between qHBsAg and HBV DNA levels in the HBeAg-negative patients.^29^

In conclusion, according to the first results of our follow-up, NA treatment can be discontinued in CHB patients without advanced/end-stage liver disease as recommended by the current hepatitis B guidelines. According to our observations, although there was no risk of decompensation or mortality, most patients experienced relapse and treatment should be reinitiated. However, after at least 24 months of follow-up, 17/57 (%29) are still in an inactive state. Older age, female gender, higher pretreatment HBV-DNA and ALT level, and higher pretreatment liver biopsy HAI score predict the relapse. All patients with HBsAg loss are the ones with previous LAM resistance that needs to be further investigated.

## Figures and Tables

**Table 1.** Characteristics of Included Patients at the Time of Nucleos(t)ide Analog Cessation (n = 57)

		Value
Age (years)^†^		49 (29-72)
Female patients, n (%)		24 (42%)
Follow-up period (months)^†^		27 (24-30)
Treatment duration (months)^†^		96 (36-276)
Consolidation therapy duration (months)†		88 (31-273)
Time to achieve undetectable HBV-DNA level (months)^†^		6 (1-28)
Previously (PEG-IFN) therapy, n (%)		6 (10%)
NAs therapy	First line (tenofovir, entecavir), n (%)	49 (86%)
	NA switch, n (%)	16 (28%)
Body mass index (kg/m^2^)^†^		26.8 (18-40.8)
Pretreatment liver biopsy (n 57) HAI^†^		6 (2-13)
Pretreatment liver biopsy (n 57) F^†^		2 (0-3)
FIB-4^†^		0.92 (0-2.18)
APRI^†^		0.209 (0-0.9)
PAGE-B^†^		7 (−2-18)
Pretreatment HBV-DNA (log_10_ IU/mL)^†^		5.31 (3.47-8.95)
Pretreatment ALT (U/L)^†^		43 (12-154)

ALT, alanine transaminase; APRI, AST-to-platelet ratio index; HAI, Knodell histological activity index; HBeAg, hepatitis B e antigen; HBV-DNA, hepatitis B virus deoxyribonucleic acid; F, Ishak fibrosis score; FIB-4, fibrosis-4 score; NAs, nucleos(t)ide analogs; PEG-IFN, pegylated interferon; PAGE-B; platelet count, age, gender.

^†^Median (range).

**Table 2.** Logistic Regression Analysis of the Factors Affecting the Persistence of HBV-DNA >2000 IU/mL

HBV-DNA >2000 IU/mL	Univariate		Multivariate	
	OR (95% CI)	*P*	OR (95% CI)	*P*
Age*	0.981 (0.924-1.042)	0.539	0.994 (0.880-1.123)	.925
Gender	1.500 (0.464-4.851)	0.498	0.826 (0.051-13.431)	.893
BMI*	1.167 (1.006-1.353)	**0.041**	1.185 (0.943-1.490)	.145
Pretreatment liver biopsy HAI Score	0.671 (0.476-0.945)	**0.022**	0.813 (0.504-1.312)	.397
PAGE-B*	1.055 (0.929-1.200)	0.408	0.939 (0.678-1.301)	.705

The results were considered significant at *P* < .05. Significant values in bold.

BMI, body mass index; CI, confidence interval; HAI, Knodell histological activity index; HBV-DNA, hepatitis B virus deoxyribonucleic acid; PAGE-B, platelet count, age, gender; OR, odds ratio.

*Quantitative factors are the values at the time of treatment discontinuation.

**Table 3.** Comparison of the Patient Groups With and Without History of Lamivudine Resistance

		Lamivudine Resistance (Positive) (n = 13)		Lamivudine Resistance (Negative) (n = 44)		*P*
Virological relapse^+^	No	5	38.5%	14	31.8%	.655
	Yes	8	61.5%	30	68.2%	
Clinical relapse^+^	No	7	53.8%	27	61.4%	.627
	Yes	6	46.2%	17	38.6%	
HBsAg loss^ǂ^	No	9	69.2%	44	100%	**.002**
	Yes	4	30.8%	0	0.0%	
Treatment duration (months)^‡^		96 (60-228)*		84 (36-276)*		.405
Consolidation therapy duration (months)^‡^		90 (49-200)*		79 (31-273)*		.419
Age (years)^‡^		50 (40-70)*		46 (29-72)*		.121
Gender+	Female	5	38.5%	19	43.2%	.762
	Male	8	61.5%	25	56.8%	
Body mass index (kg/m^2^)^‡^		25.60 (22.70-31.18)*		26.85 (18-40.80)*		.313
Previously (PEG-IFN) therapy^ǂ^	No	11	84.6%	40	90.9%	.611
	Yes	2	15.4%	4	9.1%	
Pretreatment liver biopsy HAI^‡^		6 (2-12)*		6 (3-13)*		.908
Pretreatment liver biopsy F^‡^		2 (0-3)*		2 (0-3)*		.517
FIB-4^‡^		1.20 (0.71-1.62)*		0.89 (0.06-2.18)*		**.042**
APRI^‡^		0.16 (0.11-0.32)*		0.22 (0.08-0.90)*		.068
PAGE-B^‡^		7 (0-13)*		7 [−2 to 18]*		.767
Pretreatment HBV-DNA (log_10_ IU/mL) ^‡^		4.62 (3.50-7.17)*		5.35 (3.47-8.95)*		.110
Pretreatment ALT (U/L)^‡^		47 (17-141)*		43 (12-154)*		.725

The results were considered significant at *P* < .05. Significant values in bold.

*Median (range). ^+^Chi-square test. ^ǂ^Fisher’s exact test. ^‡^Mann–Whitney *U-*test.

ALT, alanine transaminase; APRI, AST-to-platelet ratio index; HAI, Knodell histological activity index; HBsAg, hepatitis B surface antigen; HBV-DNA, hepatitis B virus deoxyribonucleic acid; F, Ishak fibrosis score; FIB-4, fibrosis-4 score; PEG-IFN, pegylated interferon; PAGE-B, platelet count, age, gender.
